# The Invasion of Coastal Areas in South China by *Ipomoea cairica* May Be Accelerated by the Ecotype Being More Locally Adapted to Salt Stress

**DOI:** 10.1371/journal.pone.0149262

**Published:** 2016-02-11

**Authors:** Gang Liu, Yang Gao, Fang-Fang Huang, Ming-Yue Yuan, Shao-Lin Peng

**Affiliations:** 1 College of Life Sciences, Shaanxi Normal University, 710119, Xi'an, China; 2 State Key Laboratory of Biocontrol and Guangdong Key Laboratory of Plant Resources, Key Laboratory of Biodiversity Dynamics and Conservation of Guangdong Higher Education Institutes, School of Life Sciences, Sun Yat-Sen University, 510275, Guangzhou, China; Shandong University, CHINA

## Abstract

Local adaptation and phenotypic plasticity are two alternative mechanisms used by invasive plants for range expansion. We conducted a series of experiments to investigate the role of these mechanisms in the recent expansion of the invasive *Ipomoea cairica* from non-saline to salt-stressed coastal habitats. A comparison of the plant’s photosynthetic traits and construction costs across habitats was conducted through a field survey. Meanwhile, a full factorial greenhouse experiment was conducted with two ecotypes (non-saline and coastal) of *I*. *cairica* and two salinity gradients (water and 4 g L^-1^ NaCl solution) to evaluate the roles of the two strategies by comparing their main traits. The results revealed that the construction cost and *A*_*max*_ of *I*. *cairica* did not change with the habitat type. The ecotype and saline treatments, however, significantly influenced the plant growth. The non-saline ecotype (NE) generally showed higher or equal plasticity of biomass-allocation and functional traits compared to the coastal ecotype (CE). However, the fitness and biomass of the NE significantly decreased with salinity, whereas those aspects of the CE did not change. Our results indicate that the recent expansion of *I*. *cairica* into coastal areas may be accelerated by the local adaptation of the CE to salt stress. Additionally, in South China, the CE will most likely evolve adaptations to both saline and non-saline environments, which will further broaden the invasion range of *I*. *cairica* in the future.

## Introduction

Invasive species are usually characterized by their ability to colonize in a broad range of environmental conditions [1,2]. The expansion of invasive plants in the ranges where introduced usually causes problems and may even endanger the local ecosystem. However, it may help to investigate how plants establish and spread in new environments [3]. According to previous studies, "two mutually non-exclusive mechanisms", i.e. phenotypic plasticity and adaptation, play important roles in the range expansion of invasive species [4,5]. Plasticity in functional traits that are related to resource acquisition can maximize the fitness of individual plants in heterogeneous environments [6]. Phenotypic plasticity can help plants pre-empt environmental resources through morphological and physiological changes [7]. For example, compared to native plants, invasive plants can better capitalize on rich resources due to the high plasticity in their growth [8]; in addition, invasive species usually exhibit higher plasticity of biomass-partitioning traits along environmental gradients [9,10]. Hence, phenotypic plasticity is frequently thought to be an important mechanism for plant invasions [5,11].

Meanwhile, invasive species can evolve rapidly in introduced ranges [12,13] because of the novel selection pressures experienced during expansion [14,15]. Successful invasion in new areas is usually associated with increased genetic differentiation among populations [16,17]. Local adaptation to diverse environments can result in more dissimilar intraspecific phenotypes [18], and promote the evolution of different ecotypes with distinctive traits [2]. Furthermore, in response to changes in abiotic factors, the introduced populations of some species have evolved higher plasticity than their native populations in the original range [19]. Such evolution and intraspecific trait divergences, which promote the expansion of invasive species into new habitats, have increasingly been proven [20,21,22].

Coastal areas are usually difficult for non-indigenous plants to colonize because of the high soil salinity. *Ipomoea cairica*, which is native to tropical Africa, causes significant ecological problems in South China [23,24]. This species is usually observed in farmlands, forest edges, roadsides, abandoned land, and other habitats without salt stress in introduced ranges. However, recent reports suggest the species is expanding into coastal areas and may cause a successful secondary invasion [25,26]. The reason for its new expansion into salt-stressed habitats has yet to be investigated. Plant traits such as biomass partitioning and functional traits have been suggested to be useful for evaluating the invasiveness of an exotic species during its expansion [27]. Species locally adapted to coastal environments usually have traits that allow them to tolerate or avoid salt stress [19]. For example, tolerance by some specie to salinity is closely related to their increased root to shoot ratio [3,28], which is thought to improve the availability of water and nutrient resources in high salinity environments [29]. For invasive plants, higher growth traits in a novel invasion range are often suggested to be important for maintaining their aggressive expansion [27,30,31]. The total biomass and bloom mass are suitable indices for evaluating the fitness of the plants across environmental gradients [27]. Plant fitness usually declines with increasing soil salinity [32]. The photosynthetic rate, which can be inhibited by salt stress and many other stress factors, directly affects the growth rate of the plants [33]. Vigorous invaders are often characterized by high photosynthetic traits [34]. The leaf construction cost is a measure of the energy invested by plants to synthesize carbon skeletons and nitrogenous compounds [35]. This cost can be indirectly related to the efficiency of resource utilization and diminished by salt stress [36]. In stressful habitats, decreased leaf construction costs are often detected [36,37] because cheaper leaves allow a plant to produce more photosynthetic structures for the same energy cost, which maximizes the whole-plant carbon gain [38].

In this study, we first compared the field performances (photosynthetic traits and nutrient content) between invasive and native plants in non-saline and coastal salt-stressed habitats, and subsequently investigated the invasion capability of ecotypes of *I*. *cairica* by comparing the main traits of the non-saline (NE) and coastal ecotypes (CE) in a reciprocal transplant experiment. We aimed to illuminate two main questions: (1) Do the two ecotypes (CE vs. NE) of *I*. *cairica* react differently to salt stress under both natural and controlled conditions? (2) Which mechanism (phenotypic plasticity or local adaptation) promotes the new invasion of *I*. *cairica* in coastal areas?

## Materials and Methods

### Field survey

No specific permissions were required for these activities in these locations because these places were not owned by anybody and research works were allowed conventionally. The field studies did not involve endangered or protected species.

Two types and four species, including the invasive species *I*. *cairica* (Convolvulaceae) and *Mikania micrantha* (Asteraceae), and the native species *I*. *triloba* (Convolvulaceae) and *Paederia foetida* (Rubiaceae), were selected for comparison. *Ipomoea triloba* is an annual vine and the other three species are perennial vines. All the species are annual or perennial vines, and are commonly observed as companion species to each other in the field. Individuals of these species from two types of habitat, i.e., a non-saline habitat (Panyu, Guangzhou) and a coastal habitat with high soil salinity (Qi’ao Island, Zhuhai, approximately 140 km from Guangzhou), were chosen for study. At least three populations of each species were investigated on both sites (saline and non-saline habitats) except for *I*. *cairica*, for which only one coastal population was located. This population covered approximately 0.5 ha of coastal area and was very close to the tidal line with frequent saltwater intrusion. Field surveys were conducted on sunny days with similar temperatures during June and July 2012. At least three individuals of each population were chosen for photosynthetic measurements. Photosynthesis was measured under sunny conditions at a sequence of light levels (1500, 1200, 1000, 800, 400, 200, 100, 80, 40, 20, 0 μmol m^-2^ s^-1^ PPFD) using an Li-6400XT Portable Photosynthesis System with a Red/Blue LED Light Source (Li-Cor, Lincoln, NE, USA). Each leaf was acclimated for 10–25 min to 1500 μmol m^-2^ s^-1^ PPFD prior to the measurement. Light curves and maximum photosynthetic rate (*A*_*max*_, μmol CO_2_ m^-2^ leaf area s^-1^) were calculated by the software package Photosynthesis (Li-Cor, Lincoln, NE, USA).

Afterward, the leaves were collected for measurements of construction costs and elemental tests. The leaves were dried in a 60°C oven and then were ground for testing. The measurements for the construction costs were conducted following a previous study [39]. For each sample, 1 g leaf powder was burned in a 500°C muffle furnace for 6 hours. The ash content (ASH) was calculated as ash mass divided by the sample mass. The leaf caloric value was measured by a calorimeter (IKA-C2000, IKA, Germany) with 0.5 g leaf powder from each sample. Elemental analyses of the leaf carbon concentration (C, %) and leaf nitrogen concentration (N, %) were performed on a Vario EL cube elemental analyzer (Elementar Analysensysteme, Hanau, Germany).

The leaf construction cost per unit of mass (CC_mass_) was calculated using the following equation [40]:

$${\text{CC}}_{\text{mass}}=\left[\left(0.0698\times △\text{Hc}-0.065\right)\times \left(1-\text{ASH}\right)\right]+7.5\times \left(\text{k}\times \text{N}∕14.0067\right)∕\text{EG}$$ (1)

where ΔHc = Caloric value/(1-Ash content), which was the ash-free heat of combustion, k was the oxidation state of the N substrate (+5 for nitrate or -3 for ammonium), and the EG, was the growth efficiency, which was estimated to be 0.87 across species [41]. In this study, for the form of N utilized by these plant species, we calculated the CC_mass_ for all species, following a previous study, by using the mean of CC_mass_ values which were calculated using the NH_4_^+^ and the NO_3_^−^ oxidation states [39].

### Greenhouse experiment

In early August 2012, the cuttings of NE and CE of the invasive *I*. *cairica* were obtained with similar length (approximately 5 cm) and diameter (approximately 4 mm) from non-saline (Guangzhou) and coastal habitats (Zhuhai) in South China, respectively. The cuttings of the NE plants were collected from populations in Guangzhou, and those of the CE plants were collected from only one coastal population, which was in Zhuhai. The cuttings were first cultured in nursery trays. Approximately 10 days later, 20 seedlings of similar sizes (approximately 5 cm in height) of each ecotype were chosen for reciprocal transplantation. Each seedling was transplanted into a plastic pot (diameter 20 cm; height 15 cm) with a wooden trellis (2 m in height). The pots were filled with peat and soil in a 1:1 ratio. The soil had been collected from a wasteland in a non-saline area.

We separated the seedlings from each ecotype into two groups, with 10 replicates for each group; one group was watered daily with pipe water, and the other group was watered daily with 4 g L^-1^ NaCl solution. We used this salt concentration to simulate the salinity of the local sea [25,26]. After three months of plant growth, we selected at least 3 pots from each treatment for measurement of the photosynthesis. Photosynthesis was measured following the aforementioned methodology. Light curves and maximum photosynthetic rate (*A*_*max*_, μmol CO_2_ m^-2^ leaf area s^-1^) were calculated by the software package Photosynthesis. The transpiration rate (TR, mmol m^-2^ s^-1^) and apparent quantum yield for CO_2_ assimilation (Φ_CO2_, mol CO_2_ mol^-1^ quanta) were recorded automatically by the machine. The water-use efficiency (WUE, μmol CO_2_ mmol^-1^ H_2_O) was calculated as the ratio of the *A*_*max*_ to the stomatal conductance (SC, mmol m^-2^ s^-1^).

Plants were grown for approximately 20 weeks and then harvested November 10–11, 2012. The deciduous leaves of each plant were collected during the experiment. Each plant was divided into leaf, stem, root, bloom, and deciduous leaf fractions, which were dried to constant mass at 60°C, and weighed. The leaf area was measured immediately after harvest using a CI-203 laser area meter (CID Bio-Science, USA). The leaf mass ratio (LMR, g g^-1^) and root mass ratio (RMR, g g^-1^), respectively, were calculated as the ratio of the leaf mass or root mass to total mass. The specific leaf area (SLA, m^2^ g^-1^) was calculated as the ratio of leaf area to leaf mass. The extra nectar of the bloom was collected before harvest and dissolved in pure water. Then, the soluble nectar concentration was measured using a hand-held refractometer (45–05, Bellingham + Stanley, Kent, UK).

### Statistical Analyses

A general linear model (GLM) was used to evaluate the effects of the independent variables of habitat (low or high soil salinity), type (invasive or native plant), and species (nested in the type) on the dependent variable CC_mass_, *A*_*max*_, C, or N from the field survey data. A MANOVA was used for detecting the influence of ecotype and salinity treatment on the growth performance of the plants. The total mass, bloom to total mass ratio, root to shoot mass ratio (R:S), number of shoots, SLA, and nectar concentration were used as the dependent variables. An ANOVA and LSD multiple comparison tests were used to detect the trait differences (biomass allocation traits: LMR, RMR, bloom mass, R:S, leaf area, number of shoots, deciduous leaf mass, and nectar concentration; functional traits: SLA, SC, TR, Φ_CO2_, WUE, and *A*_*max*_) between the two ecotypes in each treatment. Data for the SC and TR were log_10_-transformed to make the data fit a normal distribution. According to previous studies [42,43], a significant effect of soil salinity would indicate a plastic response to salinity, and a significant effect of ecotype would indicate differentiation in traits among populations, whereas a significant ecotype×salinity interaction would suggest divergence among populations in the norms of reaction to stress factors. All analyses were conducted with SAS ver. 9.1 (SAS Institute Inc., Cary, NC, USA).

## Results

### Comparing traits by field survey

**Fig 1:**
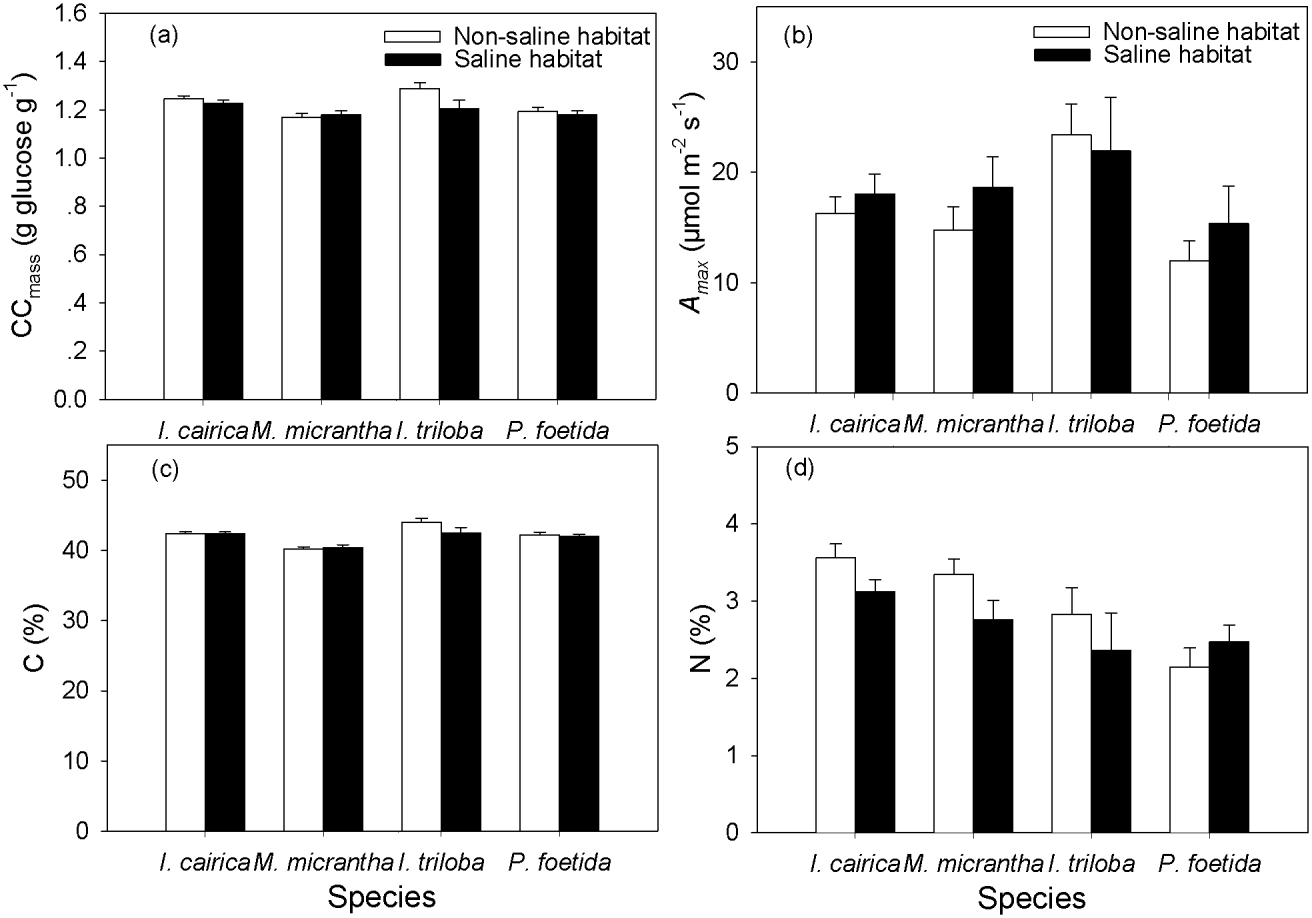
Comparisons of leaf construction cost (CC_mass_), *A*_*max*_, leaf carbon content (C), and leaf nitrogen content (N) between invasive and native species from saline or non-saline habitats. Values presented are means ± SE.

The results of the GLM revealed that the habitat type did not significantly influence the CC_mass_, *A*_*max*_, C, or N (Table 1 and Fig 1). The CC_mass_ of the invasive and native plant individuals in the highly salt-stressed habitats did not significantly increase relative to those in habitats without salt stress. Meanwhile, the *A*_*max*_ of the invasive and native plant individuals in highly salt-stressed habitats did not significantly decrease relative to those in habitats without salt stress (Fig 1). The invasive species had lower leaf carbon concentration and higher leaf nitrogen concentration compared to native species.

**Table 1:** The results of the general linear model analyses of leaf construction cost (CC_mass_), *A*_*max*_, leaf carbon content (C), and leaf nitrogen content (N).

		Habitat	Type	Habitat×Type	Species (Type)	Habitat×Species (Type)
**CC**_**mass**_	*df*	1,26	1,26	1,26	2,26	2,26
	*F*	3.53	0.66	2.52	12.39	1.4
	*P*	0.072	0.423	0.125	**0.0002**	0.264
***A***_***max***_	*df*	1,30	1,30	1,30	2,30	2,30
	*F*	0.91	0.39	0.21	3.59	0.38
	*P*	0.349	0.538	0.648	**0.04**	0.686
**C**	*F*	1.38	16.18	2.26	20.87	0.71
	*P*	0.249	**0.0004**	0.144	**<0.0001**	0.501
**N**	*F*	2.18	14.1	1.22	1.36	0.73
	*P*	0.15	**0.0007**	0.278	0.273	0.488

Independent variables were species, type and habitat. Type: invasive or native. Species: invasive species *Ipomoea cairica* and *Mikania micrantha*, and native species *I*. *triloba* and *Paederia foetida*. Habitat: saline or non-saline habitat.

### Growth performance in the greenhouse experiment

**Fig 2:**
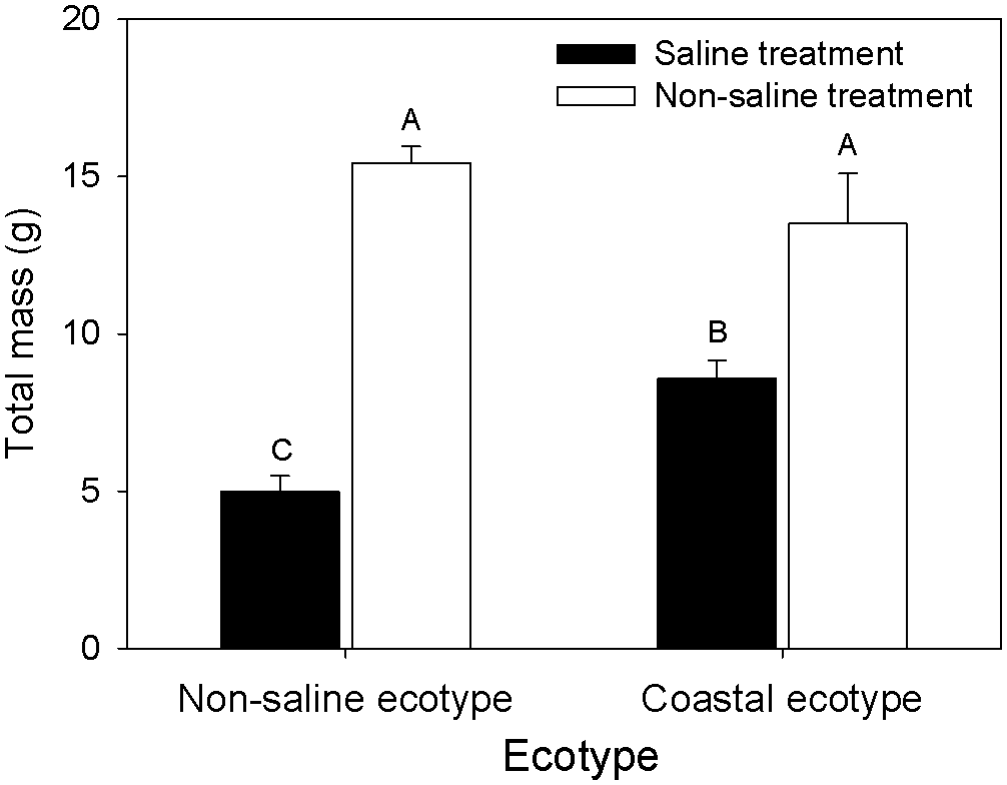
Comparison of the total mass of the non-saline and coastal ecotypes of *Ipomoea cairica* under non-saline and saline treatments. Saline treatment: 4 g L^-1^ NaCl solution; Non-saline treatment: pipe water. Different symbols indicate significant differences (α = 0.05). Values presented are means ± SE.

Both ecotype and saline treatment significantly influenced the vines’ growth performances (total mass, bloom mass/total mass, R:S, number of shoots, SLA, and nectar concentration) (Table 2). The biomasses of both ecotypes decreased significantly under salinity stress (Fig 2). However, in the saline treatment, the CE of *I*. *cairica* had higher biomass than the NE. The decrease of the CE in biomass in response to the salinity stress treatment was less than that of the NE (Fig 2).

**Table 2:** Results of the MANOVA on the traits of both ecotype of *Ipomoea cairica*.

	Wilks’ λ	*F*	num *df*	den *df*	*P*
**Ecotype**	0.173	13.588	6	17	**<0.0001**
**Saline**	0.075	35.050	6	17	**<0.0001**
**Ecotype × Saline**	0.602	1.870	6	17	0.145

Dependent variables: total mass, bloom mass to total mass ratio, root to shoot mass ratio, number of shoots, SLA, nectar concentration; factors: ecotype and saline treatment.

**Fig 3:**
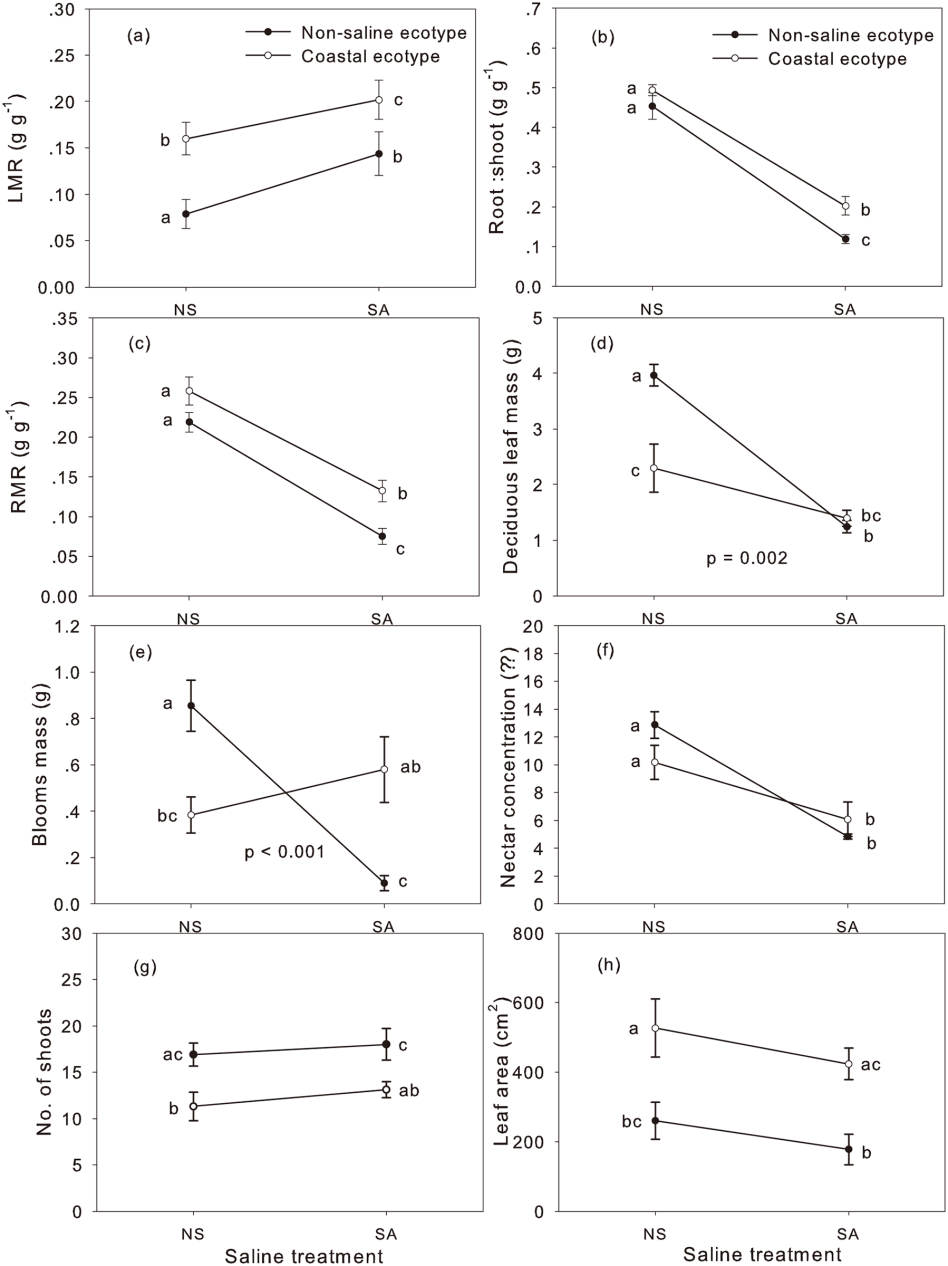
The reaction norms of the non-saline and coastal ecotypes of *Ipomoea cairica* on biomass allocation according to the saline treatment levels. NS, non-saline treatment; SA, saline treatment. LMR, leaf mass ratio; RMR, root mass ratio. The P-value reflects the statistical significance of the difference in reaction norm between the two ecotypes, which is represented by the interaction of saline treatment with ecotype (Table 3). Values presented are means ± SE. Means with the same letter were not significantly different (P < 0.05) in LSD multiple comparisons.

The biomass allocation traits were significantly influenced by the ecotype and salinity (Table 3). The LMR, RMR, R:S, bloom mass, deciduous leaf mass, and nectar concentration were significantly influenced by the saline treatments. LMR, RMR, R:S, leaf area, number of shoots, and deciduous leaf mass differed significantly between the two ecotypes. The CE had higher LMR, RMR, R:S, and leaf area, whereas the NE generally had more shoots and higher deciduous leaf mass (Fig 3 and S1 Fig).

**Table 3:** The influence of saline treatment and ecotype on the mass allocation traits.

		Corrected Model	Ecotype	Saline	Ecotype × Saline	Error
	*df*	3	1	1	1	33
**LMR**	*SS*	0.07	0.04	0.03	0.001	0.12
	*F*	6.92	12.53	7.38	0.35	
	*P*	**0.001**	**0.001**	**0.010**	0.557	
**R:S**	*SS*	0.92	0.04	0.90	0.004	0.15
	*F*	65.55	7.49	191.29	0.92	
	*P*	**<0.001**	**0.010**	**<0.001**	0.345	
**RMR**	*SS*	0.18	0.02	0.17	0.001	0.06
	*F*	32.87	11.45	89.03	0.393	
	*P*	**<0.001**	**0.002**	**<0.001**	0.535	
**Deciduous leaf mass**	*SS*	42.01	5.32	30.16	7.60	21.92
	*F*	21.08	8.01	45.40	11.45	
	*P*	**<0.001**	**0.008**	**<0.001**	**0.002**	
**Bloom mass**	*SS*	2.67	0.001	0.74	2.12	2.77
	*F*	9.00	0.01	7.48	21.40	
	*P*	**<0.001**	0.933	**0.010**	**<0.001**	
**No. of shoots**	*SS*	271.03	252.27	19.43	1.09	553.89
	*F*	5.38	15.03	1.16	0.07	
	*P*	**0.004**	**<0.001**	0.290	0.800	
**Leaf area**	*SS*	672495	601056	78603	996.32	1129621
	*F*	6.55	17.56	2.30	0.029	
	*P*	**0.001**	**<0.001**	0.139	0.866	
**Nectar concentration**	*df*	3	1	1	1	22
	*SS*	252.17	2.86	201.68	21.05	193.86
	*F*	9.54	0.32	22.89	2.39	
	*P*	**<0.001**	0.575	**<0.001**	0.136	

The reaction norms of the two ecotypes on bloom and deciduous leaf biomass allocation were generally different from each other (Fig 3). The NE showed high plasticity of the R:S, RMR, deciduous leaf mass, bloom mass, and nectar concentration, whereas the CE only showed high plasticity of the RMR and R:S (Fig 3). The NE had higher bloom mass than the CE in the non-saline treatments; however, its bloom mass significantly decreased with increasing salinity; in saline treatments, the CE had higher bloom mass than the NE (Fig 3e).

**Fig 4:**
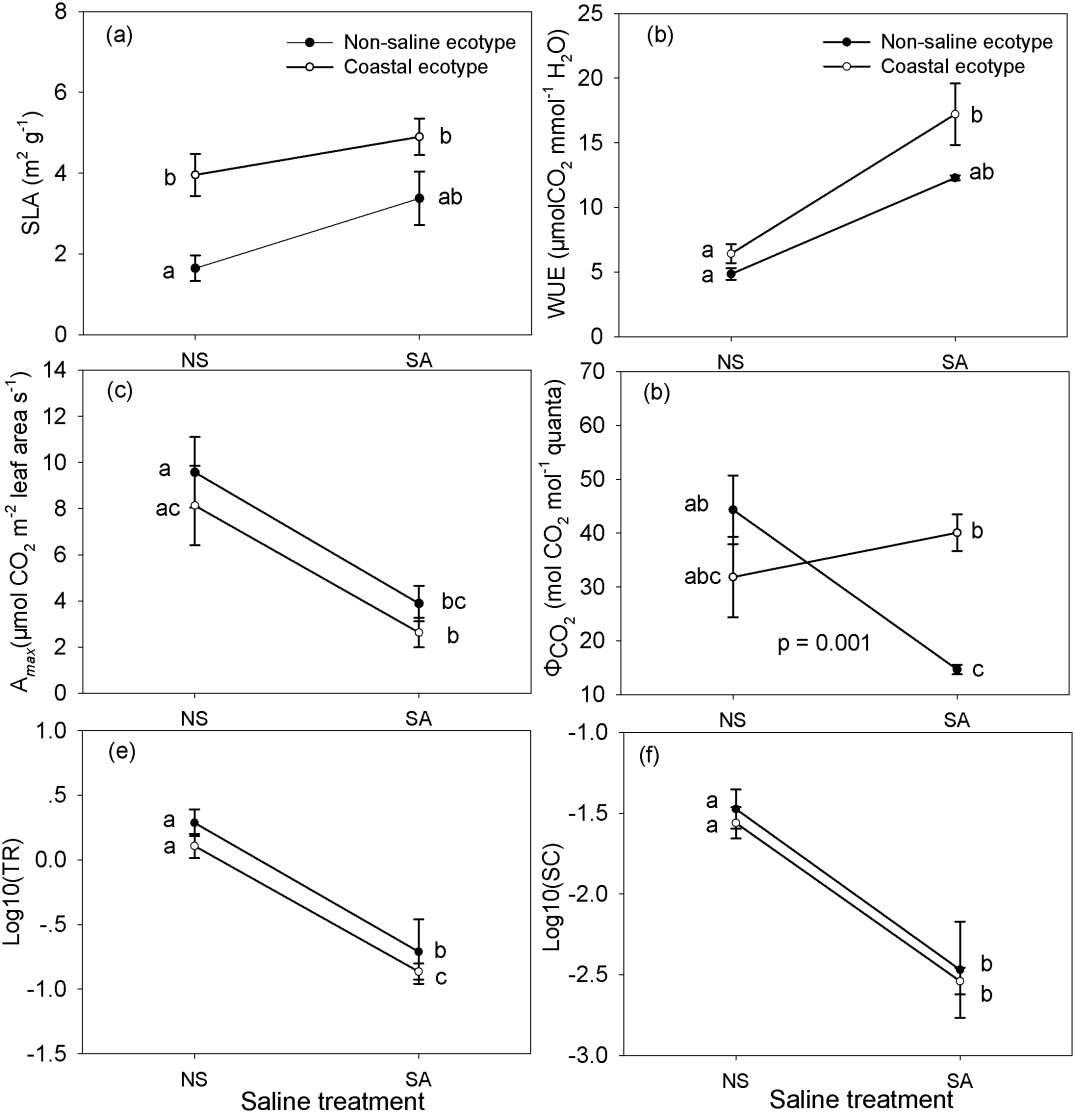
The reaction norms of the non-saline and coastal ecotypes of *Ipomoea cairica* on the functional traits according to the saline treatment levels. NS, non-saline treatment; SA, saline treatment. SLA, specific leaf area; WUE, water use efficiency; *A*_*max*_, maximum photosynthetic rate; Φ_CO2_, apparent quantum yield for CO_2_ assimilation; SC, stomatal conductance (mmol m^-2^ s^-1^); TR, transpiration rate (mmol m^-2^ s^-1^). The P-value reflects the statistical significance of the difference in reaction norm between two ecotypes, which was represented by the interaction of the saline treatment with ecotype (Table 4). Data for SC and TR were log_10_-transformed to ensure the normality of the data. Values presented are means ± SE. Means with the same letter were not significantly different (P < 0.05) in LSD multiple comparisons.

The saline treatment had a significant influence on the functional traits of the invasive species, whereas only SLA was significantly influenced by the ecotype (Table 4). The CE showed lower or equal plasticity of functional traits compared to the NE (Fig 4). The reaction norms of both ecotypes’ functional traits were similar, with the exception of Φ_CO2_. The *A*_*max*_, SC and TR of both ecotypes decreased with increasing salinity, whereas SLA and WUE generally increased with increasing salinity (Fig 4). The *A*_*max*_ of the CE and the NE decreased by 67.68% and 59.37%, respectively; their SC decreased by 89.19% and 86.22%, respectively; their TR decreased by 89.43% and 87.49%, respectively; their SLA increased by 23.90% and 105.17%, respectively; and their WUE increased by 168.26% and 153.74%, respectively.

**Table 4:** The influence of the saline treatment and ecotype on the functional traits.

		Corrected Model	Ecotype	Saline	Ecotype × Saline	Error
**SLA**	*df*	3	1	1	1	33
	*SS*	52.77	33.73	16.43	1.41	74.81
	*F*	7.76	14.88	7.25	0.62	
	*P*	**<0.001**	**0.001**	**0.011**	0.435	
**SC**	*df*	3	1	1	1	12
	*SS*	3.90	0.02	3.60	0.00	0.94
	*F*	16.66	0.29	46.08	0.00	
	*P*	**<0.001**	0.599	**<0.001**	0.956	
**TR**	*df*	3	1	1	1	12
	*SS*	4.02	0.10	3.58	0.00	0.65
	*F*	24.71	1.89	66.00	0.01	
	*P*	**<0.001**	0.194	**<0.001**	0.918	
**Φ**_**CO2**_	*df*	3	1	1	1	16
	*SS*	2739.36	259.82	628.80	1792.34	1870.29
	*F*	7.81	2.22	5.38	15.33	
	*P*	**0.002**	0.155	**0.034**	**0.001**	
**WUE**	*df*	3	1	1	1	11
	*SS*	407.7	36.19	284.95	9.62	174.6
	*F*	8.56	2.28	17.95	0.61	
	*P*	**0.003**	0.159	**0.001**	0.453	
***A***_***max***_	*SS*	127.57	6.24	107.31	0.03	47.82
	*F*	9.78	1.43	24.68	0.01	
	*P*	**0.002**	0.256	**<0.001**	0.939	

An ANOVA was used for these analyses. SLA, specific leaf area (m^2^ g^-1^); SC, stomatal conductance (mmol m^-2^ s^-1^); TR, transpiration rate (mmol m^-2^ s^-1^); Φ_CO2_, apparent quantum yield for CO_2_ assimilation (mol CO_2_ mol^-1^ quanta); WUE, water use efficiency (μmol CO_2_ mmol^-1^ H_2_O); *A*_*max*_, maximum photosynthetic rate (μmol CO_2_ m^-2^ leaf area s^-1^). The data for the SC and TR were log_10_-transformed to ensure the normality of the data.

## Discussion

Adaptation, derived from rapid evolutionary change, is important for the expansion of invaders [14]. The evolution of new phenotypes is spurred by exposure to the different selection pressures of heterogeneous environments [44]. Since the non-saline and coastal habitats mainly differ in soil salinity, we speculated that natural selection would lead to the local adaptation to high-salt habitats or variation in plasticity. In general, the CE had a higher growth performance than the NE under salt-stressed condition. This result indicates that local adaptation may play an important role in the new expansion of *I*. *cairica* into coastal areas.

In our field survey, the *A*_*max*_ and CC_mass_ of *I*. *cairica* did not change across environments. This suggests that *I*. *cairica* might maintain a stable energy balance under natural salt stressed conditions, similar to that of the widespread native species that are already well adapted to local environmental gradients. However, the result may only partly reflect the fitness of *I*. *cairica* due to the lack of direct evidence in this survey. Moreover, in this survey, the four traits of the invasive and native vines were not significantly different with each other. This probably occurs because the two native species are widespread plants with high growth traits. For instance, *P*. *foetida*, which is a native invasive species, has a photosynthetic rate and construction cost similar to that of vigorous invasive plants [39].

Reciprocal transplant experiments in a common garden are useful ways to study local adaptation and phenotypic plasticity of invasive species [2,45]. In common-garden environments, the importance of local adaptation can be evaluated by testing the divergence for important traits of invasive populations that originate across environmental gradients [43]. Species biomass allocation traits (e.g., LMR, R:S, etc.) and functional traits (SLA, photosynthetic rate, etc.) are suggested to be good indicators for evaluating invasiveness divergence [27]. The two ecotypes reacted to salt stress differently in their bloom mass, deciduous leaf mass, and Φ_CO2_ (Tables 3 and 4) owing to the significant interactions of the ecotype and salinity on them. The three traits of the CE were not significantly changed by the salt stress, whereas these three traits of the NE were decreased by the salt stress. For example, Φ_CO2_ is linearly associated with Φ_PSII_, which can be used as a good indicator of tolerance to salt stress [3,46]. The sharply decreased Φ_CO2_ of the NE indicates this ecotype’s maladaption to salt stress. Additionally, the CE generally had higher LMR, RMR, R:S, leaf area, and SLA than the NE (Figs 3 and 4). Additionally, under the salt stressed condition, the CE had higher total mass, bloom mass, WUE, and Φ_CO2_ (Figs 3, 4 and S2 Fig). The higher LMR and leaf area are associated with light reception. Mass allocation traits, particularly root mass allocation, can affect nutrient and water resource competition. High root mass usually indicates a high capacity to compete for soil nutrients and a limited water supply [47]. Plants with higher R:S are more adaptive to salt stress [3]. SLA is an indicator of photosynthetic surface area per unit investment in leaf tissue [34]. For invasive plants, higher SLA is usually correlated to high growth rate [27]. WUE is considered as a pathway by which invasive plants may increase the efficiency of resource capture [34]. In this study, the decreased R:S and increased LMR of *I*. *cairica* in response to salt stress are inconsistent with previous studies [29,48]. However, in compensation, the *I*. *cairica* showed decreased TR and SC under saline treatments (Fig 4) at the same time. Thus, the invader finally obtained increased WUE. Unlike trees and shrubs, vine species usually do not need to assign so many resources to structural support [49]. Thus, they can allocate a large proportion of their resources to produce more leaves, as well as for stem elongation and reproduction [50]. For this reason, vine species are usually more effective competitors for light relative to other plant forms [51]. Therefore, larger leaf mass should be more important than larger roots for a vine under stressful conditions. The differences in these traits suggest that the CE of *I*. *cairica* has a higher ability to tolerate salt stress than the NE. The ability of an introduced species to successfully tolerate environmental stress and invade broad geographic ranges has been suggested to mainly be related to broad environmental tolerance and local adaptation [52]. The salinity tolerance of invasive plants is important because it may govern the distribution limits of a certain plant in the field [53]. In sum, such trait divergence indicates a local adaptation of the CE to salt stress. In other words, the studied coastal population has evolved a higher ability for salt tolerance than the studied populations in the non-saline habitat. However, such a conclusion should be made cautiously at the ecotype level due to the deficiency of the sampled populations in this study.

In contrast, some species expand by means of plasticity but not local adaptation. For example, Japanese Knotweed from the salt marshes did not perform better under salt stress, suggesting that this plant is not better adapted to salt stress than those from low-salinity habitats [3]; nor did the environmental differences create genotypes of the perennial salt marsh species *Borrichia frutescens*, which was adapted to both high and low salt levels [54]. The successes of these species may be promoted by their plasticities in resource allocation, morphological characters and physiological traits, which may allow individuals to maintain high fitness across different environments [6]. Increasing empirical evidence supports phenotypic plasticity as an important mechanism for successful invasions in heterogeneous environments in local ranges [5,55,56]. Phenotypic plasticity is favored during migrations of species (even specialists) in different environments [45,57]. In this study, the CE generally showed lower or equal plasticity to the NE for most of the biomass-allocation traits (Fig 3). Apparently, the new expansion of *I*. *cairica* cannot be well explained by phenotypic plasticity. Moreover, invaders in new habitats usually exhibit greater plasticity than their conspecifics in native ranges or populations in previous ranges because of the evolving underlying plasticity [9,58]. Unexpectedly, our results demonstrated a reversed pattern, i.e., the CE showed a lower plasticity than the NE (Fig 3). Such reduced plasticity under stressful conditions has also been observed by others [59]. This pattern likely arises because strong stressful factors can lead to phenotypic integration [60], and because these traits may not be adaptive for salt tolerance. Consequently, these results imply that the expansion of *I*. *cairica* from non-saline habitats to coastal habitats should be attributed to local adaptation favored by natural selection, rather than by trait plasticity. This conclusion is in accordance with the field survey. However, differences also occur. For example, in the field survey, the *A*_*max*_ of the CE grown in the salt-stressed habitat was similar to that of the NE grown in the non-saline habitat. However, in the greenhouse experiment, the *A*_*max*_ of the NE grown in the non-saline environment was higher than that of the CE grown in the salt-stressed environment. In fact, the *A*_*max*_ is sensitive to many factors (e.g. temperature, water resource, etc.), which are often very different under wild and greenhouse conditions. The mismatch suggests caution in inferring trait reactions to stress factors under the controlled conditions.

Successful and widespread invaders are usually characterized by one of two different sets of features. Some are general-purpose species; others are specialized species adapted to certain environments [2,43]. A general-purpose ecotype invader would be expected to have high fitness across a series of environmental gradients, which shows a low variance in fitness [43]. The successful expansions of some species have been documented because of specialization in certain environments [43,61], whereas the expansion of others have proven to be favored by the superior ability to invade broad ranges [3,54,62]. The CE of *I*. *cairica* was better adapted to the high-salinity treatment than the NE. Adaptive divergence is characterized by "higher fitness of resident genotypes compared to genotypes from other habitats" [63]. The fitness of the CE, which was evaluated by total biomass and flower traits in accordance with previous research [27], was not significantly decreased by the salinity treatment, whereas that of the NE was significantly decreased (Figs 2 and 3). In this study, we did not use more suitable indices, such as seed mass or seed germination rate because *I*. *cairica* usually breeds vegetatively and rarely produces seeds. In general, the fitness of an individual is greater when its phenotype matches the environment in which it occurs [64]. For invasive plants, the maintenance of invasiveness is involved in low variance in fitness across environmental gradients; thus, a general-purpose genotype is predicted to succeed in broader ranges [65]. Consequently, the CE will most likely evolve adaptations to both the saline and non-saline environments, which will further broaden the invasion range of *I*. *cairica* in the future.

## Supporting Information

S1 Fig Comparisons of the deciduous leaf mass to total mass ratio of *Ipomoea cairica* under all treatments. NE, non-saline ecotype; CE, coastal ecotype. Values presented are means ± SE.
(TIF)

S2 Fig Comparison of the total mass (excluding the deciduous leaf mass) of the non-saline and coastal ecotypes of *Ipomoea cairica* under non-saline and saline treatments. Saline treatment: 4 g L^-1^ NaCl solution; Non-saline treatment: pipe water. Different symbols indicate significant differences (α = 0.05). Values presented are means ± SE.
(TIF)
