# Supplementary figures and images for: The Invasion of Coastal Areas in South China by Ipomoea cairica May Be Accelerated by the Ecotype Being More Locally Adapted to Salt Stress
Source: PLoS One. 2016 Feb 11;11(2):e0149262. doi: 10.1371/journal.pone.0149262 (PMC4750935; doi:10.1371/journal.pone.0149262)

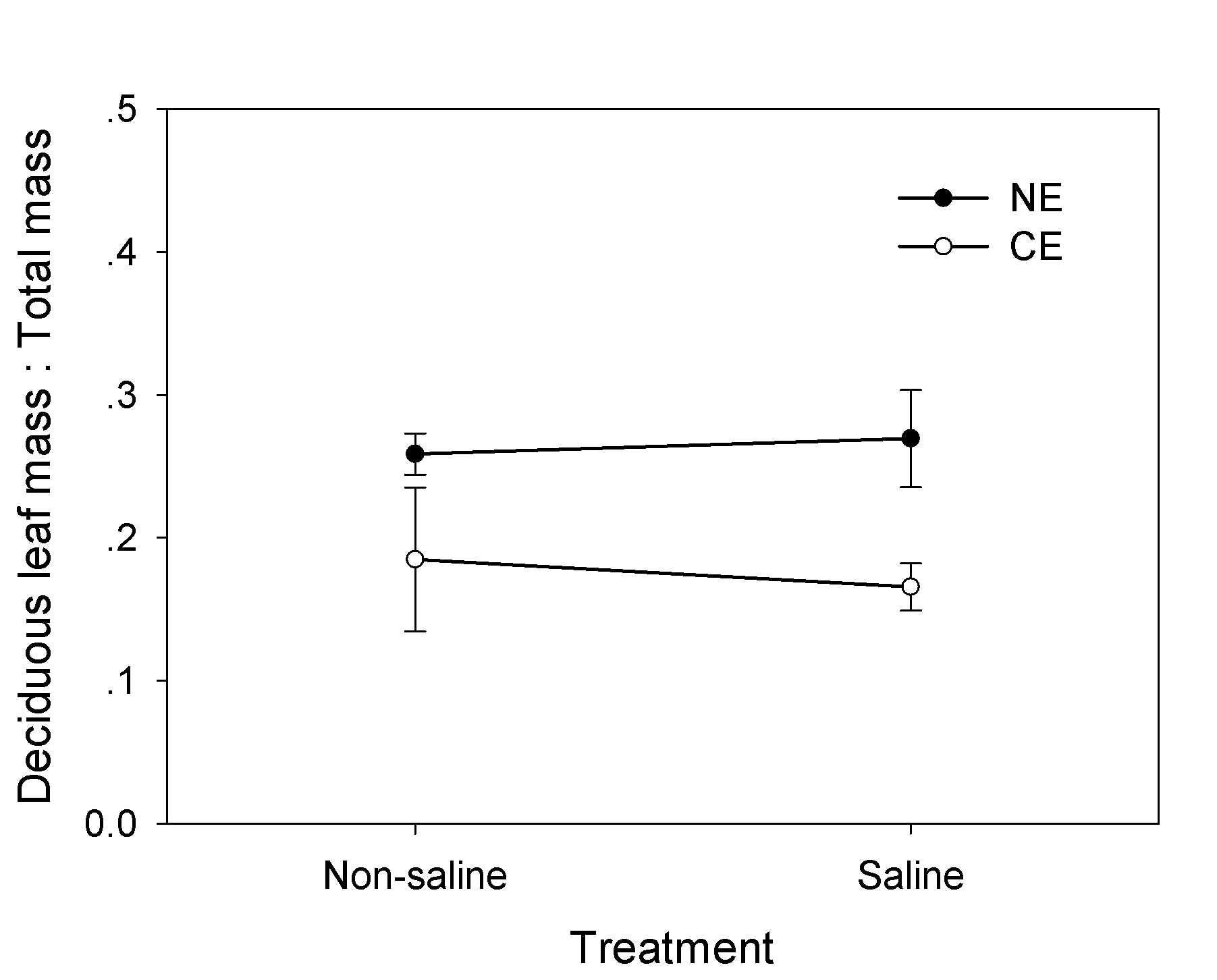

Supplement: S1 Fig — NE, non-saline ecotype; CE, coastal ecotype. Values presented are means ± SE. (TIF) [file pone.0149262.s001.tif]

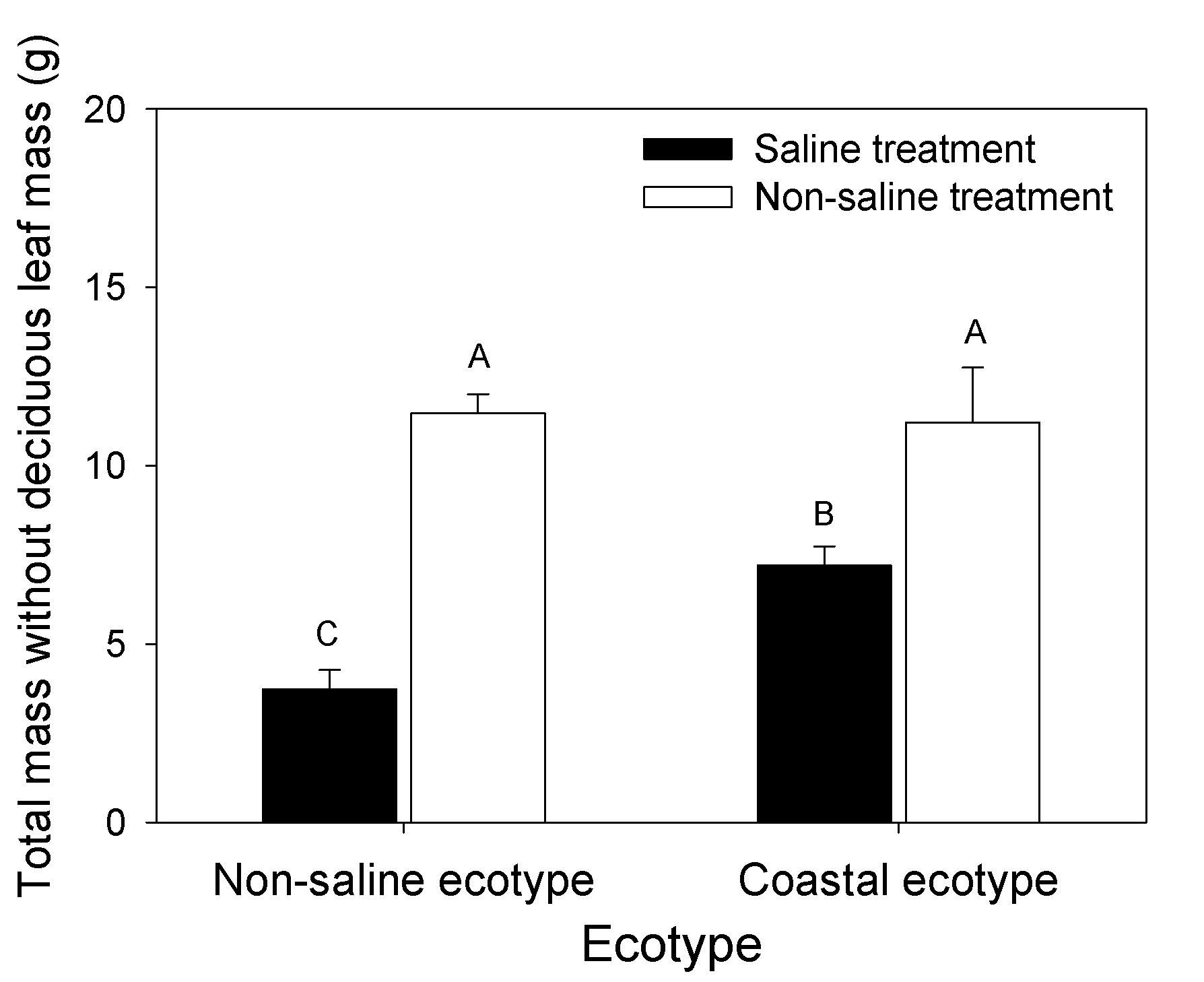

Supplement: S2 Fig — Saline treatment: 4 g L-1 NaCl solution; Non-saline treatment: pipe water. Different symbols indicate significant differences (α = 0.05). Values presented are means ± SE. (TIF) [file pone.0149262.s002.tif]
